# Bovine renal lipofuscinosis: Prevalence, genetics and impact on milk production and weight at slaughter in Danish cattle

**DOI:** 10.1186/1751-0147-51-7

**Published:** 2009-02-12

**Authors:** Jørgen S Agerholm, Knud Christensen, Søren Saxmose Nielsen, Pia Flagstad

**Affiliations:** 1 Department of Disease Biology, University of Copenhagen, Bülowsvej 17, DK-1870 Frederiksberg C, Denmark; 2 Department of Basic Animal and Veterinary Sciences, University of Copenhagen, Bülowsvej 17, DK-1870 Frederiksberg C, Denmark; 3 Department of Large Animal Sciences, Faculty of Life Sciences, University of Copenhagen, Bülowsvej 17, DK-1870 Frederiksberg C, Denmark; 4 Danish Agricultural Advisory Services, National Centre, Udkærsvej 15, Skejby, DK-8200 Århus N, Denmark

## Abstract

**Background:**

Bovine renal lipofuscinosis (BRL) is an incidental finding in cattle at slaughter. Condemnation of the kidneys as unfit for human consumption was until recently considered the only implication of BRL. Recent studies have indicated a negative influence on the health of affected animals. The present study investigated the prevalence, genetics and effect of BRL on milk yield and weight at slaughter.

**Methods:**

BRL status of slaughter cattle was recorded at four abattoirs during a 2-year-period. Data regarding breed, age, genetic descent, milk yield and weight at slaughter were extracted from the Danish Cattle Database. The prevalence of BRL was estimated stratified by breed and age-group. Furthermore, total milk yield, milk yield in last full lactation and weight at slaughter were compared for BRL-affected and non-affected Danish Holsteins and Danish Red cattle.

**Results:**

433,759 bovines were slaughtered and 787 of these had BRL. BRL was mainly diagnosed in Danish Red, Danish Holstein and crossbreds. The age of BRL affected animals varied from 11 months to 13 years, but BRL was rarely diagnosed in cattle less than 2 years of age.

The total lifelong energy corrected milk (ECM) yields were 3,136 and 4,083 kg higher for BRL affected Danish Red and Danish Holsteins, respectively. However, the median life span of affected animals was 4.9 months longer, and age-corrected total milk yield was 1,284 kg lower for BRL affected Danish Red cows. These cows produced 318 kg ECM less in their last full lactation. Weight at slaughter was not affected by BRL status.

The cases occurred in patterns consistent with autosomal recessive inheritance and several family clusters of BRL were found. Analysis of segregation ratios demonstrated the expected ratio for Danish Red cattle, but not for Danish Holsteins.

**Conclusion:**

The study confirmed that BRL is a common finding in Danish Holsteins and Danish Red cattle at slaughter. The disorder is associated with increased total milk yield due to a longer production life. However, a reduced milk yield was detected in the end of the production life in Danish Red. The study supports that BRL is inherited autosomal recessively in the Danish Red breed and Danish Holsteins, but with incomplete penetrance of the genotype in Danish Holsteins.

## Background

Bovine renal lipofuscinosis (BRL) is an incidental finding in cattle at slaughter. The disorder is due to accumulation of the pigment lipofuscin in the tubular epithelium, especially that of the proximal tubules. The accumulation is associated with brown to black discolouration of the kidneys, which are condemned as unfit for human consumption. This discolouration has given rise to the more common name "black kidney disease" [1]. Although known for more than 100 years, only a few studies have been done on this disorder and mostly focusing on the nature of the pigment [2-5]. BRL occurs in a familial pattern [6] and a recent genomic study has determined the location of the gene involved to chromosome 17 [7] independently in both breeds.

BRL was until recently considered a disorder without influence on animal health. Animals with BRL remain inconspicuous at the *ante mortem *inspection, the carcass of BRL cases are marked similar to other carcasses by the meat inspection and reports on clinical disease associated with BRL are absent worldwide. However, a recent controlled study in Danish slaughter cattle based on around 134,000 bovines indicated that BRL might have a negative influence on the health of affected animals [6]. This is not surprising as lipofuscin accumulation may occur as a result of a pathological process associated with increased damage to cellular membranes, impaired breakdown of membrane segments or both [8]. The present study aimed at evaluating: a) the prevalence of BRL stratified by age and breed; b) impact of BRL on total (lifelong) milk production; c) impact of BRL on milk yield in last full lactation; d) impact on weight at slaughter and e) familial segregation of BRL affected cattle.

## Materials and methods

### Animals

The study was carried out as a cross-sectional type study based on cattle slaughtered between September 1, 2005 and August 31, 2007 at four major abattoirs in Denmark. The abattoirs included were the Danish Crown slaughterhouses in Tønder, Aalborg and Skive and an abattoir in Herlufmagle at Zealand. These covered the same geographic regions as in our previous study [6]. However, the abattoir in Herlufmagle was used instead of NV-Ox as this had been closed and the Danish Crown abattoir in Skive was shut down by December 31, 2005.

Identification and recording of BRL cases were done by the meat inspection personnel during their routine inspection of slaughtered cattle. Recording was based on the unique compulsory eartag number, which is linked to production and pedigree data in a national cattle database. Additionally the date of slaughter and the carcass number were recorded. Recording was done manually. All other cattle slaughtered at the four abattoirs during the 2-years-period served as reference material.

### Data and analysis

The identity of BRL cases was controlled by comparing the manually recorded numbers with the data recorded in the central database on animals admitted to the abattoirs. The veterinarian in charge at the abattoir was contacted if discrepancies were found and the manual data sheet was re-examined. Discrepancies were mostly due to a simple typing error of a single digit in the 11-digit eartag number. The animals were traced by slaughter date and carcass number and the registered id-number was corrected in the dataset.

A wide range of data was extracted from the database. The type of data was basically the same as those used in our preliminary study on BRL [6]. Each set of data referred to the unique identification number and included date of slaughter, date of birth, sex, breed, sire, paternal and maternal grandsires, milk production (weight of milk, fat and protein in kg) at latest yield control and during the entire life span, lactation number at slaughter, and weight of carcass at slaughter. Except for analysis of age and breed prevalence, statistical analyses were restricted to animals ≥ 2 years of age and restricted to animals of the Danish Holstein breed or the Danish Red breed.

The analyses of the effect of BRL on milk yield were carried out in 2 steps, where the average 305 day kg energy corrected milk yield (305 day kg ECM) in the last full lactation was described for cows with and without BRL, stratified by parity (2, 3, 4 and > 5) and breed (Danish Holstein and Danish Red). The total (lifelong) kg ECM (total kg ECM) was also described for the 10 strata. Furthermore, the first, second and third quartiles of slaughter weight were described for BRL affected and non-affected animals.

Then, the difference in 305 kg ECM and total kg ECM between BRL affected and BRL non-affected was estimated by analysis of variance with BRL as a fixed effect and herd as a random effect in mixed models using the Mixed procedure in SAS v. 9.1 (SAS Institute, Cary, North Carolina, USA). The 305 day kg ECM was corrected for effect of parity in the five parity groups mentioned above. The lifelong kg ECM was assessed both with and without correction for age at slaughter to determine the effect of keeping affected animals longer.

The effect of BRL on slaughter weight was assessed by analysis of variance using a model similar to the total kg ECM. Difference in slaughter weights between BRL affected and non-affected was estimated with inclusion of herd as a random effect in a mixed effects model using the Mixed procedure in SAS v. 9.1. Only the two breeds Danish Holsteins and Danish Red were included in this analysis due to the small number of affected animals (< 5) for all other purebred animals. The analysis was also restricted to animals > 2 years of age due to the small number of reactors among young animals.

The residuals of the models were assessed to determine if they were independent, identically distributed Normal.

Statistical analysis of genealogical data was restricted to sires and grandsires having at least 100 progeny aged 2 years or older. The inheritance was evaluated by analysing the ratio between affected and unaffected progeny in families with a heterozygous sire and maternal grand sire. Only combinations with more than one affected offspring were considered and only progeny above 2 years of age were included. Segregation patterns between affected and unaffected individuals were compared to the 1:7 ratio expected for an autosomal recessive disease in the chosen breeding combination by the chi-square test.

## Results

### Basic data

A total of 433,759 bovines were admitted to the abattoirs and 787 of these had BRL. BRL was mainly diagnosed in the Danish Red and Danish Holstein breeds and crossbred animals, but a few cases were also found in other dairy breeds and beef cattle (Table 1). However, evaluation of the genetic background of these animals showed that the only 100% purebred cases were two Jersey cows while the other cases were hybrids or had an unknown or partly unregistered descent. Among adult (> 2 years of age) Danish Red, Danish Holsteins and crossbreds, prevalences were 1.3, 0.3 and 0.4%, respectively.

**Table 1:** Descriptive statistics of weight at slaughter stratified by bovine renal lipofuscinosis (BRL) phenotype and breed.

**Breed**	**Non-affected by BRL**				**Affected by BRL**			
		
	**N**	**q1**	**median**	**q3**	**N**	**q1**	**median**	**q3**
	
Danish Red	38050	212	254	300	251	251	289	327
Danish Holstein	270856	200	242	293	414	258	297	331
Danish Jersey	30366	160	188	214	4	149	167	173
Danish Red Holstein	4848	217	258	308	4	224	233.5	276.5
Finnish Ayrshire	261	212	239	267	0			
Norwegian Red	13	258	312	347	0			
Crossbred	54112	222	268	316	101	239	278	302
Jutland Cattle	22	266	277	319	0			
Simmentaler	4291	274	314	358	1	366	366	366
Swiss Brown	18	241	251	293	0			
Grauvieh	85	235	269	307	0			
Highland Cattle	219	190	230	263	0			
Danish Gelbvieh	9	203	266	310	0			
Dexter	101	146	175	193	0			
Salers	23	268	316	370	0			
Aberdeen Angus	3835	256	297	336	1	292	292	292
Galloway	319	211	237	262	0			
Hereford	7489	244	288	333	1	190	190	190
Piemontese	45	278	351	412	0			
Blonde d'Aquitaine	435	313	366	440	0			
Danish Shorthorn	84	239	280	312	0			
Danish Charolais	2778	280	324	371	0			
Limousine	9700	280	317	355	3	355	378	387
Belgian Blue	30	260	309	403	0			
Unknown	1058	245	307	330	1	331	331	331

Six affected and 3,925 unaffected animals have been excluded from the table due to unrecorded slaughter weight.

Abbreviations: q1: 1^st ^quartile; q3: 3^rd ^quartile.

Analysis of age distribution showed that most slaughter cattle were culled between the age of 6 and 24 months (54.7%). The culling profiles for the Danish Red and Danish Holstein breeds were similar with a peak around one year of age corresponding to the usual slaughter age of male calves used for fattening as previously observed [6].

The age of BRL affected animals varied from 11 months to 13 years. BRL was rarely diagnosed in cattle less than 2 years of age (n = 61) although most normal veal calves were slaughtered in this interval. Because of the obvious lack of BRL affected individuals in the young age groups, animals less than 25 months were omitted from the further statistical analyses to prevent a bias from these. Median ages at culling for BRL affected and BRL non-affected Danish Holstein and Danish Red cows included in the analyses on milk production were 54.9 and 48.7 months, respectively.

**Figure 1:**
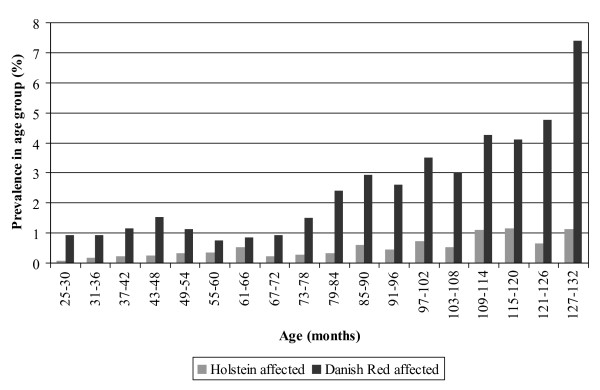
**Prevalence of renal lipofuscinosis in 6 months age groups of slaughter cattle**. The prevalence of renal lipofuscinosis increased with increasing age. Data are provided for Danish Holsteins and Danish Red cattle aged 2 to11 years.

The prevalence of BRL was estimated per six-months age intervals (i.e. for cattle aged 25 to 30 months, 31 to 36 months, etc.). The analyses were limited to cattle aged between 2 and 11 years, as BRL was infrequent in younger animals and the number of slaughter cattle aged above 11 years was very low. The calculations demonstrated a 4–5 times increase in the prevalence of BRL with increasing age for both Danish Holsteins and the Danish Red breed if the final increase for the Danish Red breed to above 7% is considered incidental (Figure 1).

### Production data

Descriptive statistics on slaughter weight and milk yield (305 day kg ECM and total lifelong kg ECM) are provided in Tables 1 and 2, respectively. Slaughter weight was not available from 3,931 animals. Results of the analyses of variance on 305 day kg ECM and total lifelong kg ECM are provided in Tables 3 and 4, respectively. The results showed that BRL affected cows of Danish Holsteins and Danish Red produced 4,083 and 3,136 total kg ECM more milk, respectively, than non-affected cows. The higher total milk yield was caused by the longer life-span of BRL affected animals (4.9 months difference between affected and non-affected animals at slaughter), and if the total kg ECM was corrected for age at culling, RBL-affected Danish Red cows produced 1,284 kg ECM less than the non-affected cows. The BRL affected Danish Red produced 318 kg ECM less in their last full lactation compared to non-affected cows (*P *= 0.0028), whereas there was no difference for Danish Holsteins (*P *= 0.7, Table 3).

**Table 2:** Descriptive statistics on milk yield for cows in 906 Danish dairy herds analysed according to bovine renal lipofuscinosis (BRL) phenotype (affected (+) *versus *unaffected (-))

		**305 day kg energy-corrected milk yield in last full lactation**						
**Breed**	**BRL**	**Parity**	**N**	**Min**	**q1**	**Median**	**q3**	**Max**

Danish Red	-	1	4414	183	6506	7451	8294	12042
	-	2	3648	1419	7292	8405	9488	13785
	-	3	2417	1169	7673	8815	9885	13827
	-	4	1298	1242	7604	8789	10045	14208
	-	> 4	1086	409	7382	8464	9626	13754
	+	1	57	3555	6053	7368	8312	10592
	+	2	30	4578	6797	8007	8810	10941
	+	3	21	4627	6843	7930	9325	10833
	+	4	26	5069	7638	8270	8929	10341
	+	> 4	33	2959	6370	7234	8576	12172
Danish Holstein	-	1	30513	167	6961	7891	8810	20315
	-	2	27074	207	7999	9126	10242	17076
	-	3	18004	150	8295	9455	10600	17737
	-	4	10264	282	8326	9524	10682	17727
	-	> 4	8644	373	8081	9260	10413	16494
	+	1	79	4410	7087	7906	8944	11266
	+	2	99	4508	7832	9273	10417	12973
	+	3	69	5868	7617	9343	10122	12506
	+	4	48	4254	8208	9349	10452	13155
	+	> 4	55	3976	8059	8960	10160	13034

	**Total (lifelong) energy-corrected milk yield**							

Danish Red	-	1	4414	183	6506	7451	8294	12042
	-	2	3648	1419	7292	8405	9488	13785
	-	3	2417	1169	7673	8815	9885	13827
	-	4	1298	1242	7604	8789	10045	14208
	-	5	1086	409	7382	8464	9626	13754
	+	1	57	3555	6053	7368	8312	10592
	+	2	30	4578	6797	8007	8810	10941
	+	3	21	4627	6843	7930	9325	10833
	+	4	26	5069	7638	8270	8929	10341
	+	> 4	33	2959	6370	7234	8576	12172
Danish Holstein	-	1	30513	167	6961	7891	8810	20315
	-	2	27074	207	7999	9126	10242	17076
	-	3	18004	150	8295	9455	10600	17737
	-	4	10264	282	8326	9524	10682	17727
	-	5	8644	373	8081	9260	10413	16494
	+	1	79	4410	7087	7906	8944	11266
	+	2	99	4508	7832	9273	10417	12973
	+	3	69	5868	7617	9343	10122	12506
	+	4	48	4254	8208	9349	10452	13155
	+	> 4	55	3976	8059	8960	10160	13034

Abbreviations: min: minimum; q1: 1^st ^quartile; q3: 3^rd ^quartile; max; maximum

**Table 3:** Estimated effect of bovine renal lipofuscinosis (BRL) on 305 day kg energy corrected milk yield in last full lactation (305 day kg ECM) of cows in two Danish dairy breeds

Danish Red	Estimate	Standard error	*P*-value
Baseline 305 day kg ECM	8107	118	< 0.0001
BRL affected	-318	106	0.0028
Parity 1	-1208	46	< 0.0001
Parity 2	-217	47	< 0.0001
Parity 3	173	49	0.0005
Parity 4	214	55	0.0001
Parity > 4	0	-	

Danish Holstein			

Baseline 305 day kg ECM	9168	80	< 0.0001
BRL affected	-29	77	0.7
Parity 1	-1414	18	< 0.0001
Parity 2	-218	18	< 0.0001
Parity 3	132	19	< 0.0001
Parity 4	217	21	< 0.0001
Parity > 4	0	-	

**Table 4:** Effect of bovine renal lipofuscinosis (BRL) on lifelong milk production (kg energy corrected milk yield (kg ECM)) in two Danish dairy breeds

Danish Red – model excluding age at slaughter	Estimate	Standard error	*P*-value
Average total kg ECM	26524	1037	< 0.0001
BRL affected	3136	1031	0.0023

Danish Holstein – model excluding age at slaughter			

Average total kg ECM	30394	814	< 0.0001
BRL affected	4083	811	< 0.0001

Danish Red – model including age at slaughter			

Baseline total kg ECM	-21,773	453	< 0.0001
BRL affected	-1284	350	< 0.0001
Age at slaughter (months)	683	2	< 0.0001

Danish Holstein – model including age at slaughter			

Baseline total kg ECM	-21,589	279	< 0.0001
BRL affected	-125	252	0.62
Age at slaughter (months)	733	0.79	< 0.0001

Weight at slaughter appeared to be higher for BRL affected animals based on the descriptive statistics (Table 1), but there was no statistical difference in weight at slaughter between BRL affected and BRL unaffected groups for neither Danish Holsteins (*P *= 0.33) nor for Danish Red cattle (*P *= 0.21, Table 5).

**Table 5:** Estimated effect of bovine renal lipofuschnosis (BRL) on weight at slaughter in two Danish dairy breeds

Danish Red	Estimate	Standard error	*P*-value
Average weight at slaughter	301	1.1	< 0.0001
BRL affected	-3.3	3.4	0.33

Danish Holstein			

Average weight at slaughter	291	0.5	< 0.0001
BRL affected	3.1	2.5	0.21

### Pedigree data

The 253 BRL cases of the Danish Red breed were progeny of 83 sires having between 1 and 39 affected progeny each, while the 417 Danish Holstein cases were progeny of 174 sires with affected progeny numbers between 1 and 77. The 312,017 unaffected animals of the Danish Red and Danish Holstein breeds were progeny of 9,520 sires with a number of progeny per sire ranging from 1 to 2,213 for sires of the Danish Red breed and from 1 to 12,616 for Danish Holsteins.

The data on affected cases were analysed for familial patterns by grouping the cases according to paternal sire. Two and three sire families were identified for the Danish Red and Danish Holstein breeds, respectively (Table 6). In addition to 17 sons having affected progeny, they also had 125 sons, which had only unaffected progeny. These sons were generally characterized by having less that 10 adult progeny each. The sire A5 in Table 6, with only 1 affected out of 133, might represent an error in the paternity of the affected animal.

**Table 6:** Number of progeny being unaffected or affected by bovine renal lipofuscinosis and aged at least 25 months.

Sire	No. of unaffected progeny	No. of affected progeny
A1	242	39
A2	69	4
A3	2	1
A4	5	2
A5*	132	1
A6	0	1
B1	167	11
B2	4	2
B3	117	9
C1	1507	49
C2	9	1
C3	16	1
C4	4	1
D1	4920	77
E1	20	1
E2	3776	13
E3	12	2

Progeny grouped according to paternal sire (A-E) and sire (1, 2, 3..). Sire A and B were of the Danish Red breed while sires C to E were Danish Holsteins

* Sire A5 might have been included due to an error in the paternity of the affected animal.

Analysis of BRL cases showed that 19 and 118 cases occurred in family clusters in the Danish Red breed (3 clusters) and the Danish Holstein breed (11 clusters), respectively. The corresponding numbers of unaffected cattle in these family clusters counted 100 and 2,261 bovines, respectively. Consequently, BRL occurred in 16% of the Danish Red cattle, which is not different from the expected 12.5% (*P *> 0.25). By contrast, only 5.0% of the Danish Holsteins were affected which is significantly less than expected (*P *< 0.0001).

## Discussion

The study confirms that BRL is a rather common disorder in adult slaughter cattle with prevalences of 0.3 and 1.3 in Danish Holsteins and Danish Red cattle, respectively. Brown discolouration of the kidneys was also found in other breeds (Table 1), but the aetiology of the pigment in these has not been investigated. Most of these cases were in breed hybrids or had an unregistered descent, so they might be genetically related to a breed known to harbour the defect. However, purebred Jersey cows with brown discoloured kidneys were found, so BRL or a disorder with a similar morphology occur in this breed. Further studies are needed to clarify the nature of the pigment and pathogenesis of brown discolouration of the kidneys in other breeds than the Danish Holstein and Danish Red.

BRL has for many years been a common finding at *post mortem *examination of slaughter cattle, so the veterinary meat inspection staff is generally considered to be familiar with the morphology. The ability to diagnose the disorder was not evaluated as part of this study, but 100% specificity has been found previously [6]. The specificity is also considered very high in the present study although other disorders may be associated with dark discolouration of the kidneys. Pigments as haemoglobin, myoglobin and porphyrins may accumulate in the kidneys. However, this occurs as a consequence of a primary disease as i.e. acute haemolytic anaemia or congenital erythropoietic porphyria [9]; diseases that are most likely recognised at the *post mortem *inspection. The sensitivity of the *post mortem *diagnostic has not been studied, but very low-grade discolouration may remain unrecognised. However, experienced meat inspection personnel is generally believed to have excellent skills in recognising renal discolouration as they see many normal kidneys each day (the kidneys of more than 400,000 bovines were examined during this study) and as the kidneys of all carcasses must be examined. The frequency of reporting BRL in Danish Red cattle and Danish Holsteins aged at least 25 months at each abattoir was examined (data not shown). Significant differences were found but these were not systematic, i.e. one abattoir had a significant higher reporting rate in Danish Red, but a similar rate in Danish Holsteins. Some cases probably remained unreported due to errors in the registration procedure, i.e. if members of the permanent staff were replaced shortly due to illness or vacation, but such events were considered to be random events, which could occur at all four abattoirs independent of occurrence of BRL. The observed prevalences were slightly lower than previous found and they probably represent minimum prevalences. The differences in reporting rates among the abattoirs may reflect differences in the BRL gene frequency in regional cattle populations.

As previously reported [6] BRL mainly occurred in adult animals. However, it was shown that the prevalence of BRL increased with age. This might suggest that the lipofuscin accumulation is an aging phenomenon as for lipofuscin accumulation in neurons and cardiomyocytes. However, the cells of the renal tubular epithelium do not belong to a stable cell population as neurons and cardiomyocytes do, but to a cell population with regular turnover. The turn-over time for proximal tubular cells is not known for cattle, but it has been demonstrated that 0.6% of the cells in the proximal tubular segment S3 of adult rats enters cell cycle each day [10,11]. An age dependent histomorphological recognisable increased amount of lipofuscin is therefore not expected unless the turnover rate decreases with increasing age of the animal. That the lipofuscin accumulation is not a simple aging phenomenon is supported by the observation that the degree of discolouration is age independent [6]. A simple age dependent accumulation would most likely be expressed as slight discolouration in younger animals and severe discolouration in older animals. This is not the case. The increased prevalence rather indicates that the BRL genotype is mainly expressed in older animals and that BRL affected animals are maintained in the herds for a longer period than unaffected individuals.

The effect of BRL appeared to affect the milk yield in Red Danish, but not in Danish Holsteins (Tables 3 and 4). However, the longer lifespan of BRL affected animals required that age was included in the analyses, further complicating the interpretation. The analysis of the total kg ECM demonstrated that the lifelong milk production in BRL affected cattle was higher, but that the higher milk yield was due to a longer lifespan. If affected cows had been culled at an age comparable to the age of non-affected cows, they would have produced significantly less milk (Table 4). The weight at slaughter did not appear to be affected by BRL (Table 5), although the descriptive results suggested so (Table 1). A likely explanation is the effect of herd, which is not apparent from the descriptive statistics. The effect of BRL on milk production was not observed in our original study [6], probably because the number of cases was too low. The reduction was not due to a lower breeding value of the sires, but was probably associated with BRL. Similar observations regarding production loss were not done for the Danish Holstein breed (Tables 3 and 4). At present the reasons for this remain speculative. One explanation could be that the disorder is due to different mutations within the same gene, with different functional implications. Alternatively, there might be differences between allelic variants in modifier genes within the two breeds. That BRL in Danish Holstein and Danish Red cattle is expressed in a different way has previously been indicated by a different degree of renal discolouration between the breeds [6]. Genomic studies are needed to investigate this aspect further.

A previous study has demonstrated that BRL is an inherited trait in Danish Holstein and Danish Red cattle. Cases occurred in family clusters and affected cattle occurred in frequencies consistent with autosomal recessive inheritance in that study if the Hardy-Weinberg criteria were assumed fulfilled [6]. The segregation ratio between BRL affected and unaffected progeny was examined in the present study in families with a heterozygous sire and maternal grand sire. The ratio between affected and unaffected progeny should equal 1:7 for autosomal recessively inherited disorders. Analysis of segregation ratios in 3 such clusters in Danish Red cattle corresponded to the expected ratio thus confirming previous findings of autosomal recessive inheritance. However, affected and unaffected progeny in 11 clusters of Danish Holsteins did not segregate 1:7. This could indicate that BRL is not inherited autosomal recessively in Holstein, but a more likely explanation is that BRL is expressed in a lower rate in Danish Holsteins or at a more advanced age due to incomplete penetrance of the genotype. This hypothesis is supported by the fact that simple Mendelian inheritance has been confirmed in both breeds by SNP-based association mapping providing a unique, genome-wide significant signal at BAT17 [7]. Identification and characterization of the causative mutation at BAT17 will clarify the phenotypic differences between breeds. It is not surprising that interpretation of segregation ratios was problematic. Evaluation of progeny phenotypes is often difficult when the phenotype is expressed at different ages, especially if it is expressed at an advanced age because some animals may not reach that age before being eliminated from the population.

## Conclusion

The study confirms that BRL is a common finding in Danish Holsteins and Danish Red cattle at slaughter. BRL affected animals generally have a longer life span, which can result in a higher total lifelong milk production. However, the disorder was associated with reduced milk yield for Danish Red cattle in the last lactation and in age-span corrected total milk yield, while affected Danish Holsteins seem to have normal production values. The longer life span may not be due to an actual prolonged production period in BRL affected cows (i.e. due to positive selection), but rather reflect that the risk of having BRL increases with increased age. It was confirmed by analysis of segregation ratios that BRL is inherited autosomal recessively in the Danish Red breed. Similar findings were not made for Danish Holsteins, probably because of a different age profile of affected animals.

## Competing interests

The authors declare that they have no competing interests.

## Authors' contributions

JSA made the study design, coordinated the sampling of BRL data, participated in interpretation of the results and drafted the manuscript. KC and SSN performed the data analyses and data interpretation. PF extracted data from the Central Cattle Database. All authors read and approved the final manuscript.
